# Visualization of DNA Damage and Protection by Atomic Force Microscopy in Liquid

**DOI:** 10.3390/ijms23084388

**Published:** 2022-04-15

**Authors:** Tinghui Dai, Yanwei Wang, Guangcan Yang

**Affiliations:** Department of Physics, Wenzhou University, Wenzhou 325035, China; 194511082198@stu.wzu.edu.cn

**Keywords:** peroxynitrite, ectoine, imaging in liquid, DNA damage, persistence length, single-stranded DNA, protective influence

## Abstract

DNA damage is closely related to cancer and many aging-related diseases. Peroxynitrite is a strong oxidant, thus a typical DNA damage agent, and is a major mediator of the inflammation-associated pathogenesis. For the first time, we directly visualized the process of DNA damage by peroxynitrite and DNA protection by ectoine via atomic force microscopy in liquid. We found that the persistence length of DNA decreases significantly by adding a small amount of peroxynitrite, but the observed DNA chains are still intact. Specifically, the persistence length of linear DNA in a low concentration of peroxynitrite (0 µM to 200 µM) solution decreases from about 47 nm to 4 nm. For circular plasmid DNA, we observed the enhanced superhelices of plasmid DNA due to the chain soften. When the concentration of peroxynitrite was above 300 µM, we observed the fragments of DNA. Interestingly, we also identified single-stranded DNAs during the damage process, which is also confirmed by ultraviolet spectroscopy. However, if we added 500 mM ectoine to the high concentration PN solution, almost no DNA fragments due to double strand breaks were observed because of the protection of ectoine. This protection is consistent with the similar effect for DNA damage caused by ionizing radiation and oxygenation. We ascribe DNA protection to the preferential hydration of ectoine.

## 1. Introduction

DNA damage has emerged as a primary culprit in cancer and many diseases related to aging [1]. In fact, the integrity of genomic DNA is constantly under threat because of DNA lesions, even in perfectly healthy cells [2,3]. These lesions can block genome replication and transcription, and if they are not repaired or are repaired incorrectly, they lead to mutations or wider-scale genome aberrations that threaten cell or organism viability [4]. Furthermore, defects in DNA damage-induced apoptosis contribute to tumorigenesis and the resistance of cancer cells to a variety of therapeutic agents [2,5]. One main source of DNA is environmental factors, such as ultraviolet light, ionizing radiation, and numerous genotoxic chemicals [3]. Among these chemicals, peroxynitrite (PN) is a strong oxidant, thus is a typical DNA damage agent, produced by immune cells, and is closely related to many inflammation-associated pathogeneses. However, the pathogenic mechanism of PN is still not fully clear and is needed to be further explored extensively. In the present study, we try to investigate the interaction between PN and DNA to obtain some insights for developing new methods to cure related diseases. 

PN is toxic and mobile among cells [6,7,8,9,10], and thereby, in vivo, PN is implicated in many diseases, including neurodegenerative and cardiovascular disorders, inflammation, cancer, and aging [11,12]. There is now substantial evidence that PN can impair most cellular components, and potential biological targets of PN include membrane as well as cytosolic and nuclear receptors [6,13]. Under physiological conditions, the PN anion becomes partly protonated (pKa = 6.8) to form PN acid [14,15]; many biomolecules are damaged by PN-derived radicals due to its strong oxidative and nitrifying feature [6,7]. Especially, PN has a significantly detrimental impact on the DNA molecule, causing strand breaks by attacking the DNA phosphate backbone, modifying bases, and deoxyribose oxidation, that consequently causes mismatches and induces mutations [7,16,17,18,19,20]. However, structural alteration in DNA induced by PN is still not fully known [21].

In contrast, antioxidants such as vitamin E (tocopherol, tocotrienol) and catalase (CAT) provide electrons to free radicals to neutralize them since these compounds can accommodate the loss of an electron without becoming reactive [22]. Ectoine in particular has good oxidation resistance and a role as a hydroxyl radical scavenger [23,24,25]. Ectoine is synthesized and accumulated in molar concentration by bacteria to withstand osmotic stress [26,27]. Even at a high molar concentration, ectoine and related substances do not disturb the metabolic pathways within the cell and are therefore called compatible solutes [28]. Ectoine water structuring effects [29,30,31], its role as a hydroxyl radical scavenger [23,24] and DNA melting temperature [32,33], DNA structure [34,35,36], and protection of biomolecules against ionizing radiation [24] and oxidative stress [23,28] were investigated over the last decade. Due to its versatile properties, ectoine is currently used in a multitude of biotechnological, cosmetic, and medical applications. In some studies, the antioxidative efficacy of ectoine in in vitro models was evaluated. Ectoine reduces the release of certain inflammatory factors, and thus it was postulated that ectoine diminishes DNA damage in inflammatory processes caused by PN radiation.

PN-induced DNA damage and the corresponding protection were studied by a few groups using different methods in recent years. For example, as early as 1998, Stephen C. Grace et al. discovered that scavenging of peroxynitrite by a phenolic/peroxidase system prevents oxidative damage to DNA by UV-Vis and agarose gel electrophoresis [37]. Goutam Chowdhury et al. discovered the protective role of glutathione against PN-mediated DNA damage during acute inflammation by UV-Vis spectra and fluorescence spectroscopy [38]. G. Anushree et al. systemically carried out to decipher the changes induced by peroxynitrite using UV-Vis spectra, circular dichroism, molecular dynamics simulation followed by restriction digestion [39]. M.A. Schröter et al. found that ectoine protects DNA from damage by ionizing radiation, and compelling experimental results were obtained with AFM [28]. They deduced the single and double strand breaks of DNA based on the results of agarose gel electrophoresis. However, we still do not have a clear picture of the process of DNA structure changes caused by PN at different concentrations and the protective role of ectoine in this damaging process. In the present work, we used liquid phase AFM to obtain the conformational changes of DNA in a physiological environment containing various concentrations of PN and ectoine. Specifically, we used AFM to figure out DNA conformation changes by many physical and chemical factors, including the type of counterions, pH in solution, and hydrophobicity of agents [40,41,42,43,44,45].

## 2. Results

### 2.1. AFM Characterize the DNA Damage Process

**Figure 1:**
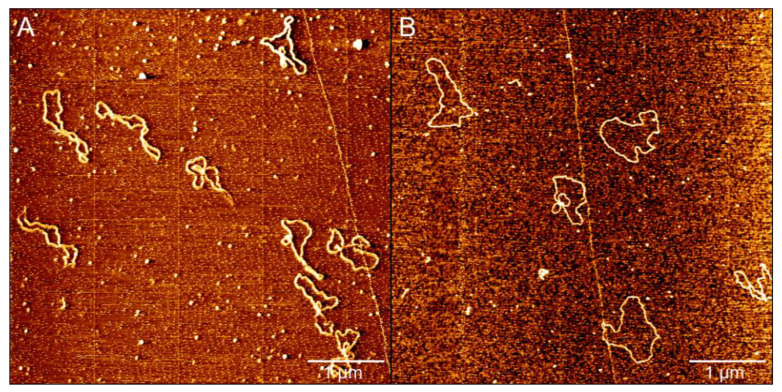
(**A**) AFM images of pBR322 plasmids in deposition buffer (25 mM KCl, 10 mM MgCl_2_, 10 mM HEPES (pH 7.5)). (**B**) AFM images of pBR322 plasmids in deposition buffer (1 mM MgCl_2_, 10 mM HEPES (pH 7.5)) by syringe pump, and the flow rate is 2 min/mL.

AFM has been known to be a powerful tool for determining some subtle changes in DNA conformation. We used AFM to investigate the variation of DNA morphology in the presence of different concentrations of PN in solution. Two types of DNA were used; one is circular plasmid pBR322 DNA(4361 bp), and the other is linear 4000 bp marker DNA. We used the standard protocol [46,47] to obtain AFM images of DNA but with some modification to have more loosely natural plasmid DNAs. The main modification includes: 1. Reducing the concentration of MgCl_2_ in deposition buffer from 10 mM to 1 mM. 2. Using the syringe pump to exchange liquid at a constant and slow speed. As shown in Figure 1, we demonstrated a comparison of AFM images obtained by the original and modified protocols. We can see the supercoils of plasmid DNA in Figure 1A by the standard protocol. In contrast, we can notice that the circular DNA becomes more as loose loops as shown in Figure 1B, because of the flow of liquid in the process of DNA deposition on the mica surface. We infer that the slow exchange of fluid in the sample cell hinders the formation of supercoils of DNA, although the internal strain of circular DNA is still maintained due to the link number conservation of DNA topology. 

**Figure 2:**
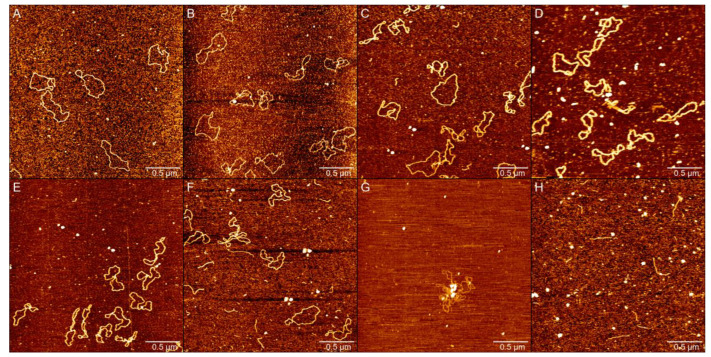
AFM images of pBR322 plasmids in deposition buffer (1 mM MgCl_2_, 10 mM HEPES (pH 7.5)) at different concentrations of PN. (**A**) Without PN. In (**B**–**H**), the concentration of PN is 50 µM, 100 µM, 150 µM, 200 µM, 300 µM, 400 µM, 1 mM.

The impact of PN on plasmid DNA is shown in Figure 2, in which the AFM images of circular plasmid DNA on the mica surface in liquid are presented at various concentrations of PN solution. For the negative control, we start by imaging DNA alone in the same buffer condition as used in the presence of PN, as shown in Figure 2A, which shows the typical circular conformation of DNA in the absence of PN as expected. From Figure 2B to (H), the corresponding PN concentrations in solution increase gradually from 50 µM to 1 mM. We can see a dose-dependent change in the conformation of DNA. 

In Figure 2A, we found that in the absence of PN, the plasmid DNA showed complete natural loops or with one crossing. When 50 µM PN is added to the solution, as shown in Figure 2B, we found that more than 1/2 of plasmid DNA produces kinks. Meanwhile, we can see more supercoils of DNA in the image. As shown in Figure 2C,D, when the concentration of PN reaches 100 µM to 150 µM, the number of nicks increases and the proportion of DNA that produces kink gradually increases; moreover, the number of kinks on single DNA increases, indicating that the degree of DNA distortion also further increases, and DNA becomes softer. In Figure 2E, when the concentration of PN reached 200 µM, almost all plasmid DNA produced kink, and the proportion of DNA that produces kinks reached the maximum. When the concentration of PN reached 300 µM, the plasmid DNA in Figure 2F was double-stranded broken, and the circular structure was damaged, resulting in linear DNA fragments and entanglement among different DNA. We found that the entangled plasmid DNA had more kinks and was softer than the unentangled plasmid DNA. As shown in Figure 2G, when the concentration of PN reached 400 µM, all plasmid DNA produced kinks and were entangled with each other, similar to slight DNA aggregation. Finally, we increased the concentration of PN to 1 mM in Figure 2H; almost all of the pBR322 DNA becomes linear DNA, and the amount of DNA is significantly reduced on the mica.

We can ascribe the increase in supercoils kinks of DNA to the increasing nicks of single strand break caused by PN. The increasing nicks make the double strand DNA softer before the whole loop breaks; that is, its persistence length decreases with the concentration of PN. Before the loops of DNA are completely broken, single strand breaks release out the internal strain of the DNA loops, leading to the formation of supercoils of DNA because of the conservation of link numbers, as seen in the AFM images.

**Figure 3:**
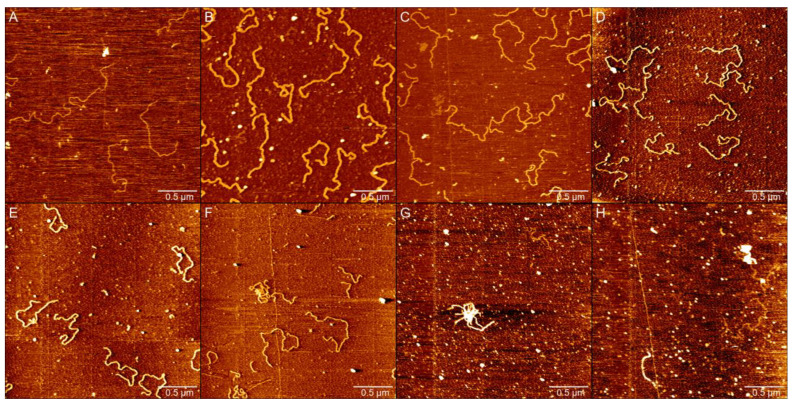
AFM images of 4000 bp linear DNA in deposition buffer (1 mM MgCl_2_, 10 mM HEPES (pH 7.5)) at different concentrations of PN. (**A**) Without PN. (**B**–**H**) The concentration of PN is 50 µM, 100 µM, 150 µM, 200 µM, 300 µM, 400 µM, 500 µM.

To explore the influence of PN on DNA further, we imaged 4000 bp linear marker DNA in liquid at various PN concentrations. The obtained AFM images are presented in Figure 3 listed in sequence of PN concentration in solution. In the case of the absence of PN, DNA is naturally extended on the fresh mica surface, as shown in Figure 3A. When the concentration of PN increases from 50 µM to 200 µM, as shown in Figure 3B–E, we can see that the DNAs have more bends and turns than those in Figure 3A. Furthermore, the number of bends and turns increases monotonically with PN concentration. This observation is similar to the case of circular DNA aforementioned. The difference is that no supercoils can be observed because of the release of internal strain for linear DNA. In Figure 3F, as the concentration of PN increased to 300 µM, some of the DNA became condensed, and the yield of DNA fragments was caused by a double strand break increase. When the concentration of PN reaches 400 µM in Figure 3G, almost all of the DNA becomes condensed, and tiny DNA fragments caused by double strand break were found. When the concentration of PN reaches 500 µM in Figure 3H, the amount of DNA on the mica is very small; there are only a few DNA fragments formed caused by double strand break.

AFM images of both circular and linear DNA demonstrate the damage of DNA by PN. According to previous studies, exposure of pBR322 DNA to 50 µM PN converted more than 90% of the native supercoiled (SC) form to a relaxed open circular (OC) form with single strand breaks [37]. As expected, we can see that most the DNAs are relaxed open circular (OC) in Figure 2B; their conformational differences are relatively small; we can see that open circular (one strand nicked) pbr322 DNA has more bends compared with those in Figure 2A, but few kinks can be found, and almost no DNA is entangled with each other. As shown in Figure 2C,E, at the low concentration of PN in 100 µM to 200 µM, the DNA has almost no observed double strand breaks; the effect of PN on DNA is only to make DNA more flexible; writhing increases with PN; the degree of plectonemic supercoiling in the plasmid molecule increased until, in the plasmid molecules, most of the duplex segments became a plectonemically supercoiled structure. In Figure 2F, as the concentration of PN increased to 300 µM, on the one hand, the yield of a linearized (LIN) form caused by double strand break increases; we are starting to see significant DNA double-strand breaks; on the other hand, some DNA starts to get tangled up. When the concentration of peroxide nitrite reaches 400 µM in Figure 2G, almost all of the DNA is stuck together, and inexplicably, little linear DNA was found. Finally, we increased the concentration of PN to 1 mM in Figure 2H; almost all of the pBR322 DNA becomes linear DNA, and the amount of DNA is significantly reduced on the mica.

The configuration changes in plasmid DNA compared with 4000 bp linear DNA in different concentrations of PN show basically the same trend, but plasmid DNA is much more curved than linear DNA in the concentration of PN from 50 µM to 200 µM. To investigate the influence of PN (50 µM to 200 µM) upon the flexibility of DNA quantitatively, we measured the end-to-end distances of linear DNA fragments and calculated their persistence lengths with the wormlike chain (WLC) model. The sharpness of AFM tips is related to the measurement of the width of DNA from sample to sample. However, this does not affect our analysis of the conformational change of DNA and the measurement of persistence length significantly. According to the worm-like chain model, we can estimate the persistence length from the mean square end-to-end distance of DNA by atomic force microscopy in liquid. The relation between them is as follows [48]:

$${\langle R^{2}\rangle }_{3D}=2L_{p}L\left(1-\frac{L_{p}}{L}\left(1-e^{-\frac{L}{L_{p}}}\right)\right)$$

where 〈R^2^〉 is the mean square end-to-end length of the polymer; L is the original contour length; L_p_ is the persistence length. By inserting the mean square end-to-end distance (〈R^2^〉) of DNA at various PN concentrations into the expression, we obtain the corresponding persistence length (L_p_) of DNA, respectively, shown Table 1. 

The mean square root distances of linear DNA fragments are presented in Table 1. We can see that the mean square root distance of DNA decreases monotonically with an increasing PN (0 µM to 200 µM) concentration. For example, the mean square root end-to-end distance of 4000 bp linear DNA is 863 nm in the absence of PN in solution, while it decreases drastically to 720 nm in the presence of 100 µM PN in solution. It means a very low concentration of PN has an impact on the change in the end-to-end distance of DNA, and thus on the persistence of DNA. When the concentration goes up to 200 µM, the mean square root end-to-end distance decreases to 373 nm. This indicates that a high concentration of PN has a much stronger damage effect on DNA than a low concentration of PN. For example, as the concentration of PN increased to 200 µM, the initial persistence length (L_p_) of a bare DNA of 47 nm decreased and reached 4 nm.

### 2.2. UV Spectroscopy in the Process of DNA Damage by PN

**Figure 4:**
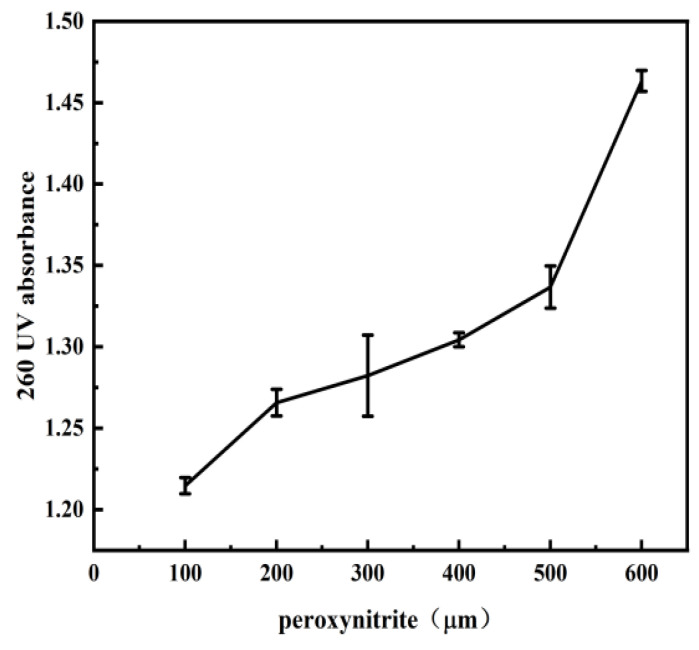
A total of 260 nm absorbance of DNA at different concentrations of PN.

To confirm further the effect of single strand break in DNA by peroxynitrite, we used ultraviolet spectroscopy to measure the hyperchromicity of DNA solution with or without PN. The hyperchromicity of DNA occurs when the DNA duplex is denatured or when the two single DNA strands are being separated partly or wholly [49]. We measured UV absorbance at 260 nm of DNA (50 µM−1 mM) solution containing various concentrations of PN, as shown in Figure 4. We can see that absorbance increases monotonically with the concentration of PN. The observation indicates the DNA structural distortion, which is either due to the opening of the double helix or single stranded breaks. These spectroscopic data confirm the mechanism of single strand break further and are consistent with the observation by AFM images in previous sections.

### 2.3. Ectoine Protects DNA against PN-Mediated Strand Breaks

**Figure 5:**
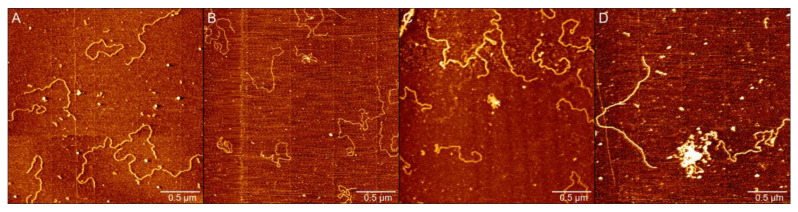
AFM images of 4000 bp linear DNA in deposition buffer (1 mM MgCl_2_, 10 mM HEPES (pH 7.5)) which contain 500 mM ectoine at different concentrations of PN. (**A**) 300 µM, (**B**) 400 µM, (**C**) 500 µM, (**D**) 1 mM.

Preservation of DNA is a prerequisite for proper cell function and faithful transmission of the genome to progeny. Ectoine is a cosolute and osmolyte, and is also a protectant of biomolecules such as protein and DNA in cells against environmental stress such as salinity, freezing, drying, and high temperatures. To investigate the protection effect of ectoine to DNA damage by peroxynitrite, we observed the change in DNA morphology in PN solution with or without ectoine by AFM, as shown in Figure 5. In the protocol, linear DNA of 4000 bp was dissolved in deposition buffer (1 mM MgCl_2_, 10 mM HEPES (pH 7.5)) containing ectoine (500 mM) and exposed in PN (300 µM, 400 µM, 500 µM, 1 mM). As shown in Figure 5A, DNA showed no condensation or double strand breaks compared with Figure 3F. As the concentration of PN increases to 400 µM, some DNA becomes condensed, but we observed almost no DNA fragments caused by double strand breaks. Comparing Figure 5C with Figure 3D, we found that the length of DNA remained normal, and the amount of DNA is very high under the protection of ectoine. Finally, we directly increased the PN concentration to a high concentration of 1 mM in Figure 5D; we can still see long double strands of DNA, but the concentration of PN is too high; some DNA has condensed and appears to be breaking into some short fragments. The protective effect of ectoine appears to have reached its limit. From the direct observation, we can see the significant protection of DNA of ectoine from the damage of PN-mediated strand breaks.

### 2.4. Strand Breaks and Single-Stranded DNA Found Induced by Peroxynitrite

**Figure 6:**
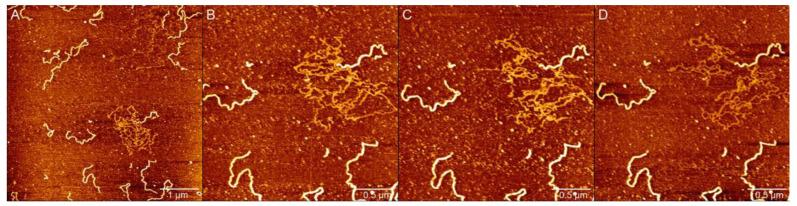
AFM images of 4000 bp linear DNA in deposition buffer (1 mM MgCl_2_, 10 mM HEPES (pH 7.5)) with 300 µM PN. (**A**–**D**) Images of repeated scanning on the same area for single stranded DNA.

Interestingly, we observed single-stranded DNA in the process of DNA damage induced by PN. Usually, it is hard to use AFM to observe ssDNA on the usual deposition substrates for dsDNA since the ssDNA forms many secondary structures due to intra-strand base-pairing. However, when 300 µM PN is added into the solution, ssDNA and dsDNA molecules are well adsorbed and can be easily identified, as shown in Figure 6A, in which we can see that a lot of ssDNA is tangled together. In the figure, we can find out that ssDNA appears quite dim in contrast to the bright dsDNA since the height of ssDNA appears to be much lower than dsDNA on the mica surface. Specifically, for dsDNA, its height and width are about 0.8 nm and 16 nm, respectively. In addition, the corresponding values of ssDNA are about 0.3 nm and 7 nm, respectively. On the other hand, the deposition of ssDNA on the mica surface is much weaker than dsDNA and can be perturbed by AFM probes. We conducted AFM repeated continuous imaging of ssDNA in Figure 6A. As shown in Figure 6B–D, dsDNA morphology was very stable with almost no change in morphology, while ssDNA underwent certain morphological changes under the force of the needle tip, presenting a different morphology in multiple imaging. In addition, after we reduced the probe force, ssDNA showed a no-pull image. Compared with dsDNA, ssDNA is more flexible and has a weaker adsorption capacity for mica. 

## 3. Discussion

**Figure 7:**
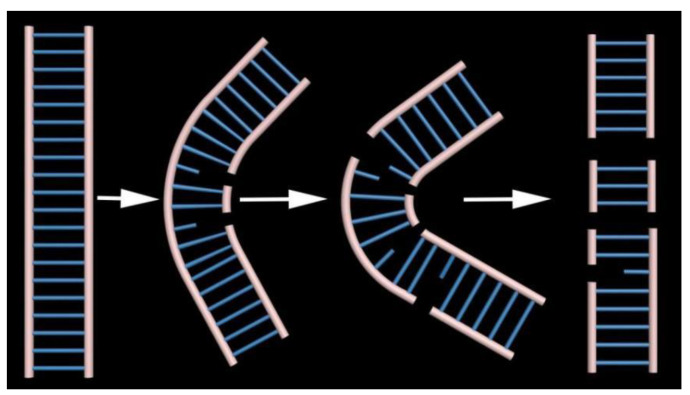
Damage process of PN to DNA.

Based on the data by AFM and spectroscopy, we proposed a possible molecular mechanism on the interaction between PN and DNA to explain the phenomenon, as shown in Figure 7 schematically. In the mechanism, PN attacks DNA, resulting in the oxidation of deoxyribose or formation of 8-Nitro-Guanine, being labile, easy to generate a single strand nicked, ultimately resulting in single strand breaks [9,50]. With the concentration of PN increasing, the number of single strand nicks increases, and the compact DNA double helix structure suffers varying degrees of damage. This allows the DNA to be more flexible, leading to a tangled and slightly compressed structure. When the nicks between the two strands are at the same position, or two SSBs arise very closely, double-strand breaks (DSBs) are formed [4]. 

A broad range of naturally occurring compatible solutes is proven to protect or stabilize proteins or other bio-molecules [51]. Although the molecular details of the underlying mechanism are still not fully understood, most of the explanations attribute the resulting protein stabilization mechanism to a preferential exclusion of cosolute around the macro-molecular compounds [35,36,52]. Ectoine shows a zwitterionic form in aqueous solution, with a carboxylic group bearing the negative charge and a partially mesomeric structure for the location of the positive charge in the pyrimidine ring. Since DNA itself has a pronounced negative charge at the backbone, the occurrence of strong electrostatic interactions between DNA and the positively charged groups of ectoine is favored. However, in our experiment, this strong binding is not so effective because of the presence of at least millimolar concentrations of Mg^2+^ in the solution, which neutralizes most of the charge of the phosphate diester backbone. Therefore, we believe that the preferential exclusion mechanism of exocrine around DNA is still effective under high ionic strength conditions. In this regard, the cosolute molecules that are repelled from the immediate vicinity of the protein surface are successively replaced by excess water molecules, which stabilize the native form in terms of a preferential hydration mechanism. We hypothesized that this stable water shell could be interpreted as an effective barrier against the interaction between PN and DNA, promoting the protection of DNA.

**Figure 8:**
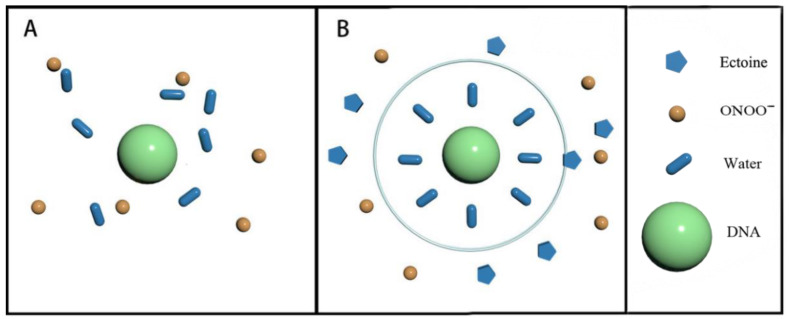
Schematic representation of interactions between DNA and ONOO^−^ in ectoine solution. The central circle denotes DNA, blue oval as water, small orange circle as ONOO^−^, and pentagon as ectoine. (**A**) interactions between DNA and ONOO^−^; (**B**) interactions between DNA and ONOO^−^ in presence of ectoine.

The DNA protection mechanism by ectoine can be explained schematically in Figure 8 based on a preferential exclusion of cosolute around the macro-molecular compounds. Ectoine shows a zwitterionic form in aqueous solution, with a carboxylic group bearing the negative charge and a partially mesomeric structure for the location of the positive charge in the pyrimidine ring. Since DNA itself has a pronounced negative charge at the backbone, the occurrence of strong electrostatic interactions between DNA and the positively charged groups of ectoine is favored. The cosolute molecules that are repelled from the immediate vicinity of the DNA surface are successively replaced by excess water molecules, which stabilize the native form of DNA in terms of a preferential hydration mechanism. This stable water shell is an effective barrier against the PN approaching DNA. Figure 8A shows the case of mild counterion concentration without ectoine in solution. In this case, the water shell around the DNA is not very organized, and ONOO^−^ is scattered around the DNA. As shown in Figure 8B, when the external base is added to the solution, the water shell becomes more organized due to the preferential rejection of the cosolute, forming a protective shell that blocks the interaction between DNA and PN, thus achieving the protective effect.

## 4. Materials and Methods

### 4.1. Materials

Chloride salts of sodium, magnesium, and nickel were purchased from Sigma-Aldrich. DNA (pBR322 plasmid and linear 4000 bp) was purchased from Fermentas (Thermo Fisher Scientific, Inc., Waltham, MA, USA), and its initial concentration was 500 ng/µL. PN was purchased from Cayman CHEMICAL. Calf thymus DNA and ectoine (1, 4, 5, 6-tetrahydro-methyl-4-pyrimidinecarboxylic acid, purity ≥ 95.0%) were purchased from Sigma-Aldrich (Sain Louis, MO, USA) and were used without further purification. Purified water was obtained from a Milli-Q system (Millipore, Billerica, MA, USA). HEPES buffer (10 mM) was used as both stock solution and experimental buffer solution. All chemical agents were used as received, and all measurements were repeated at least three times for consistency.

### 4.2. UV-Analysis of DNA Treated with PN

Denaturation (hyperchromicity) of DNA was analyzed by UV–Vis spectra. An appropriate amount of the calf thymus DNA was dissolved in 1 mM PBS buffer and stored at 4 °C as a DNA stock solution. The final DNA concentration was fixed at 60 ng/µL. Then, the diluted DNA was treated with different concentrations of PN ranging from 50 µM to 1 mM. The PBS buffer solution containing 0 µM PN without DNA was used as a blank control. After 30 min of reaction between PN and DNA at 25 °C, 2 µL of the prepared solution were loaded onto the pedestal of the Q5000 Ultra-micro ultraviolet spectrophotometer (Quawell, San Jose, CA, USA) for absorbance measurement at 260 nm.

### 4.3. Sample Preparation for AFM Imaging

DNA (500 ng/µL) stock was prepared and diluted to 1:500 (*v*/*v*) with deposition buffer (1 mM MgCl_2_, 10 mM HEPES (pH 7.5)), and the diluted DNA was treated with different concentrations of PN at the specified concentration. After a 30 min incubation at 25 °C, we deposited the sample onto mica based on a recently developed protocol [46].

As a brief outline of the process, mica (1 × 1 cm^2^) was fixed to a liquid cell (20-mm-diameter, 5-mm-height) via epoxy (Ted Pella, 16,218), cleaved with masking tape. We then deposited 20 µL of the DNA mixture onto the surface, waited for 2 s, and then serially rinsed the surface with 10 mL of deposition buffer (1 mM MgCl_2_, 10 mM HEPES (pH 7.5)) by a syringe pump, and the flow rate is 2 min/mL. Keep 500 µL of liquid in a liquid cell during rinsing. Finally, we rinsed the sample with 2 mL of imaging buffer (10 mM NiCl_2_, 10 mM HEPES (pH 7.5)) in the same way.

### 4.4. AFM Imaging

We imaged all samples on a commercial Nano Wizard III AFM (JPK Instruments AG, Berlin, Germany) with a liquid cell. After loading, the sample and cantilever settled for at least 15 min before imaging. Images were obtained in tapping mode with a typical setpoint amplitude of ~2 nm and a free amplitude of 150% of the set point. We chose the drive frequency as the closest peak of the drive transfer function to the thermal resonance when measured ~1 µm above the surface. Images of 512 × 512 pixels were acquired at a 1 Hz scan rate. The standard 2.5 × 2.5 µm^2^ images were obtained using a Bruker SNL-10A cantilever (r_nom_ ≈ 2 nm; k_typ_ = 350 pN/nm) with a 13 kHz resonance in liquid.

In AFM experiments, three replicates per condition were deposited on mica, and at least three different parts of each sample were imaged. For each experimental condition, not fewer than 9 AFM images on 2–3 mica samples were captured. By doing so, typically, several hundred DNA plasmids were included in the analysis for each sample. A visual assessment of each image was carried out by two independent persons to count the amounts of different plasmid structures. 

Full contour length, local denaturation length, long axis, short axis, and end-to-end distance measurements of DNA molecules were performed by the software Image J (Wayne Rasband, National Institute of Health, Bethesda, MD, USA), as shown in Figure 3. Each measured DNA was manually traced through the mapping tool of the software and was traced at least three times, and the length was automatically measured by the software. The average value of the three times was taken. We analyzed approximately 25 imaged DNA samples for each concentration group.

## 5. Conclusions

In summary, we directly demonstrated the process of DNA damage and protection by atomic force microscopy in liquid. By analyzing the AFM images quantitatively, we found that peroxynitrite induces a single strand break of DNA, resulting in shortened persistence length and more compact DNA morphology, finally leading a double strand break in DNA. Specifically, a high concentration of PN can decrease DNA persistence length to about one-tenth of its normal value before completely breaking. We also observed the production of single stranded DNA aggregates during the process of DNA damage by PN. However, the addition of ectoine in about a molar concentration can effectively reduce DNA double-strand breaks and protect the integrity of DNA chains. 

## Figures and Tables

**Table 1:** Root-mean-square end-to-end distance of 4000 bp linear DNA at different concentrations of PN.

Concentration of PN (%)	$\sqrt{\langle R^{2}\rangle }\;(\mathrm{nm})$	L_p_ (nm)
0	863 ± 37	47 ± 6
50	758 ± 47	32 ± 5
100	720 ± 55	26 ± 5
150	441 ± 67	9 ± 4
200	373 ± 103	4 ± 1

## Data Availability

The data presented in this study are available in the article.
